# Monitoring patients with juvenile idiopathic arthritis using health-related quality of life

**DOI:** 10.1186/s12969-021-00527-z

**Published:** 2021-03-22

**Authors:** Martijn J. H. Doeleman, Sytze de Roock, Nathan Buijsse, Mark Klein, Gouke J. Bonsel, Vicki Seyfert-Margolis, Joost F. Swart, Nico M. Wulffraat

**Affiliations:** 1 Department of Pediatric Immunology and Rheumatology, Wilhelmina Children’s Hospital, University Medical Center Utrecht, P.O. Box 85090, 3508 AB Utrecht, The Netherlands; 2 Faculty of Medicine, Utrecht University, Utrecht, The Netherlands; 3 The EuroQol Research Foundation, Rotterdam, The Netherlands; 4 Department of Public Health, Erasmus Medical Center, Rotterdam, The Netherlands; 5 MyOwnMed Inc., Bethesda, MD USA

**Keywords:** Juvenile idiopathic arthritis, Health-related quality of life, EQ-5D-Y-5 L, Disease activity, Remote monitoring, Mobile applications, E-health

## Abstract

**Background:**

Pediatric patients with juvenile idiopathic arthritis (JIA) are at risk for a lower health-related quality of life compared to their healthy peers. Remote monitoring of health-related quality of life using electronic patient-reported outcomes could provide important information to treating physicians. The aim of this study was to investigate if self-assessment with the EuroQol five-dimensional ‘youth’ questionnaire with five levels (EQ-5D-Y-5 L) inside a mobile E-health application could identify JIA patients in need of possible treatment adjustments.

**Methods:**

The EQ-5D-Y-5 L was completed via a mobile application (Reuma2Go) between October 2017 and January 2019. The clinical juvenile arthritis disease activity score with 71 joint count (cJADAS-71) was reported at every corresponding visit as reference for disease activity. Previously described cJADAS-71 thresholds were used to identify patients in possible need of treatment adjustments. Discriminatory power of the EQ-5D-Y-5 L was assessed by ROC-curves and diagnostic characteristics.

**Results:**

Sixty-eight JIA patients completed the EQ-5D-Y-5 L questionnaire. Median cJADAS-71 indicated low disease activity overall in the studied population. ROC curves and diagnostic characteristics demonstrated that self-assessment with the EQ-5D-Y-5 L could distinguish between patients with inactive disease (or minimal disease activity) and moderate to high disease activity with good accuracy (87%), sensitivity (85%), specificity (89%) and negative predictive value (86%).

**Conclusions:**

Results demonstrate that the EQ-5D-Y-5 L was able to identify JIA patients in need of possible treatment adjustments in our studied population. Remote monitoring of health-related quality of life and patient-reported outcomes via E-health applications could provide important additional information to determine the frequency of clinical visits, assess therapeutic efficacy and guide treat-to-target strategies in pediatric patients with JIA.

**Supplementary Information:**

The online version contains supplementary material available at 10.1186/s12969-021-00527-z.

## Background

Over the past two decades, the use of biologic therapies has substantially improved disease control and outcome for patients with juvenile idiopathic arthritis (JIA). Nevertheless, patients with JIA are still at risk for a significantly worse health-related quality of life (HRQoL) when compared to their healthy peers [1–4]. Persisting pain and fatigue, recurrent disease activity, and impaired societal participation remain issues for many patients with JIA [5–7]. This emphasizes the importance of adequately monitoring HRQoL.

Although several patient-reported outcomes (PROs) include measures for HRQoL, such as the Pediatric Quality of Life Inventory Rheumatology Module version 3.0 [8] and the Juvenile Arthritis Multidimensional Assessment Report (JAMAR) [9], most of these measures are extensive and require clinical visits. Therefore, frequent application required for detailed monitoring can be difficult, especially with pediatric patients.

In contrast to these disease-specific measures, the EuroQol five-dimensional ‘youth’ questionnaire (EQ-5D-Y) is a generic measure of HRQoL that takes approximately 1–2 min to complete and has demonstrated to be a valid and feasible instrument to monitor HRQoL in children and adolescents with JIA and other chronic conditions [10–12]. Recently, the EQ-5D-Y has been extended by the EuroQol Group to a five-level classification system (EQ-5D-Y-5L), hereby reducing ceiling effects and improving discriminatory power [13–16].

In order to facilitate remote monitoring and frequent self-assessment of PROs, we have recently developed a mobile E-health application, called “Reuma2Go”. The mobile application includes the EQ-5D-Y-5L as measurement of HRQoL. Since current treat-to-target guidelines recommend incorporation of PROs and HRQoL in patient assessment and therapeutic decisions [17], the present study aimed to investigate if remote self-assessment with the EQ-5D-Y-5L could identify JIA patients in need of possible treatment adjustments.

## Methods

### Study design and participants

The study was designed as a retrospective monocentric study using pseudonymized data from the Reuma2Go application and electronic health records, and was performed in accordance with the Declaration of Helsinki. This study did not fall under the scope of the Medical Research Involving Human Subjects Act, as declared by the locally appointed ethics committee at our hospital (no. 16-361C).

Patients with a diagnosis of JIA according to the International League of Associations for Rheumatology classification criteria [18] visiting our hospital between October 2017 and January 2019 were included in the study. Patients were included regardless of age, JIA subcategory, disease duration, disease activity or therapy. Informed consent for the Reuma2Go application was obtained from all parents and children of 12 years and older. Standard practice was not influenced by data from the Reuma2Go during the study period.

### Study instruments

A mobile version of the EQ-5D-Y-5L was completed in the Dutch language on the Reuma2Go application (See Supplementary Figures S1-S7, Additional File 1). The EQ-5D-Y-5 L is a five-level classification system to measure HRQoL consisting of five domains on mobility, self-care, daily activities, pain/discomfort, and anxiety/depression [13]. Each domain can be scored on a five-level ordinal scale: 1 = ‘no problems’, 2 = ‘slight problems’, 3 = ‘moderate problems’, 4 = ‘severe problems’, 5 = ‘extreme problems / inability to perform function’. Finally, the respondent is asked to rate his/her current health on a visual analogue scale (VAS) ranging from 0 = ‘the worst health you can imagine’ to 100 = ‘the best health you can imagine’ (EQ-VAS) [19]. Patients with inactive disease (cJADAS range 0–0.1) scoring level 1 = ‘no problems’ on every domain of the EQ-5D-Y-5 L accompanied by the lowest possible EQ-VAS, indicating ‘the worst health you can imagine’, were excluded from analysis because we could not be certain if they misinterpreted the EQ-VAS. The participant’s response can be conveniently summarised by an unweighted sum of the individual level scores (EQ-5D sum score) ranging from 5 to 25; or an overall EQ5D-utility score (further discussed elsewhere [15]). Responses were analysed when completed within two weeks prior to a clinical visit without any event in between. The first eligible EQ-5D-Y-5L response and its respective visit was included for patients who completed multiple questionnaires at multiple visits. In the current version of the Reuma2Go application it is not registered who completes the questionnaires (parent or patient).

As part of standard clinical care, the clinical juvenile arthritis disease activity score with 71 joint-count (cJADAS-71) is reported by the treating pediatric rheumatologist at each clinical visit [20]. The cJADAS-71 is a composite measure of disease activity and consists of the sum of its three components: an active joint count (AJC); a physician’s global assessment of disease activity (PGA), measured on a 0–10 VAS where 0 = ‘no activity’ and 10 = ‘maximum activity’; and a patient/parent assessment of overall well-being, measured on a 0–10 VAS where 0 = ‘best’ and 10 = ‘worst’.

In line with current treat-to-target guidelines and previously proposed cJADAS-71 cut-off values corresponding to a disease activity state of moderate to high disease activity, patients in need of possible treatment adjustments were defined as cJADAS-71 > 1.5 and > 2.5 for oligoarthritis and polyarthritis, respectively [17, 21].

### Statistical analysis

Baseline characteristics were analysed using descriptive statistics. Included patients were divided into two groups according to cJADAS-71 cut-off values as described above. Differences in proportions between groups were examined by Fisher’s exact test. Mann-Whitney U was used to test differences for continuous variables, as appropriate. To test for statistically significant differences between proportion of reported problems, EQ-5D-Y-5L responses were dichotomized into ‘no problems’ or ‘any problems’. For the primary objective, discriminatory power of the EQ-5D-Y-5L was examined by computing Receiver Operating Characteristic (ROC) curves and area under the curve (AUC) for each individual EQ-5D-Y-5L domain, the EQ-VAS and the unweighted EQ-5D sum score. Optimal threshold were selected using Youden’s Index [22] and further analysed by calculating accuracy, sensitivity, specificity, positive predictive value, and negative predictive value. All statistical analyses were two-sided with *p*-values < 0.05 considered as statistically significant. Analyses were performed using R version 3.5.1 with packages ‘pROC’ version 1.16.1 and ‘caret’ version 6.0–85 [23].

## Results

During the study period, 72 patients completed the EQ-5D-Y-5L questionnaire via the Reuma2Go application within a median of 0 days (IQR 0–4) prior to their clinical visit. Four patients with inactive disease scoring ‘no problems’ on every level of the EQ5D, while also scoring the highest EQ-VAS being “the worst health you can imagine” were excluded from the analysis because we could not be certain if they misinterpreted the EQ-VAS. Most of the remaining 68 patients were female (69%) and were diagnosed with persistent oligoarticular JIA (40%). Disease duration was significantly shorter in JIA patients with moderate to high disease activity according to the specific cut-off values for oligo and polyarticular JIA (*p-value = 0.01*). Characteristics of all included patients are presented in Table 1.

**Table 1:** Characteristics of included patients

	Total	cJADAS-71 < =1.5 or < =2.5^a^	cJADAS-71 > 1.5 or > 2.5^b^
**Patients, n (%)**	68	35	33
**Female, n (%)**	47 (69)	24 (69)	23 (70)
**JIA Subcategory, n (%)**			
***Oligoarticular JIA persistent***	27 (40)	12 (34)	15 (45)
***Oligoarticular JIA extended***	6 (9)	3 (9)	3 (9)
***Polyarticular JIA RF-***	15 (22)	9 (26)	6 (18)
***Polyarticular JIA RF+***	5 (7)	1 (3)	4 (12)
***Psoriatic Arthritis***	7 (10)	6 (17)	1 (3)
***Enthesitis Related Arthritis***	5 (7)	2 (6)	3 (9)
***Systemic JIA***	1 (2)	1 (3)	0 (−)
***Undifferentiated JIA***	2 (3)	1 (3)	1 (3)
**Age at visit, median (IQR), y**	13.6 (10.5–16.4)	13.3 (11.0–15.6)	14.1 (9.0–16.9)
**Age at disease onset, median (IQR), y**	7.6 (3.1–12.1)	6.7 (3.0–11.2)	9.4 (3.4–13.6)
**Disease duration, median (IQR), y**	4.6 (1.5–7.7)	6.4 (2.7–8.4)	2.4 (1.2–6.3)
**cJADAS-71, median (IQR)**	1.8 (0.1–6.1)	0.1 (0.0–0.7)	6.5 (4.0–9.5)
**Treatment at Visit**			
**Biologic DMARD, n (%)**			
***Adalimumab***	17 (25)	10 (29)	7 (21)
***Etanercept***	8 (12)	4 (11)	4 (12)
***Golimumab***	4 (6)	0 (−)	4 (12)
**Synthetic DMARD, n (%)**			
***Methotrexate***	30 (44)	17 (49)	13 (39)
***Other DMARD***	5 (7)	3 (9)	2 (6)
**No Biologic or Synthetic DMARD n (%)**	24 (35)	12 (34)	12 (36)

cJADAS-71: clinical juvenile arthritis disease activity score with 71 joint count; DMARD: disease modifying anti-rheumatic drug; IQR: interquartile range. ^a^Low disease activity for oligoarticular or polyarticular JIA, respectively. ^b^ Moderate or high disease activity for oligoarticular or polyarticular JIA, respectively

EQ-5D-Y-5L responses were without missing values. The proportion of patients reporting ‘no problems’ vs. ‘any problems’ was significantly different for all five EQ-5D-Y-5L domains between the two patient groups (*p-values < 0.001*), as was the difference in EQ-VAS responses and total EQ-5D sum score (Table 2*)*.

**Table 2:** EQ-5D-Y-5L responses of included patients

	Total	cJADAS-71 < =1.5 or < =2.5	cJADAS-71 > 1.5 or > 2.5
**EQ-5D-Y-5L Domains**	*n* = 68	*n* = 35	*n* = 33
Q01 Mobility (Walking)*			
**No problems**	44	34	10
**Slight problems**	13	1	12
**Moderate problems**	5	0	5
**Severe problems**	6	0	6
**Unable**	0	0	0
Q02 Self-Care (Washing or Dressing)*			
**No problems**	54	35	19
**Slight problems**	10	0	10
**Moderate problems**	4	0	4
**Severe problems**	0	0	0
**Unable**	0	0	0
Q03 Daily Activities*			
**No problems**	33	27	6
**Slight problems**	22	8	14
**Moderate problems**	7	0	7
**Severe problems**	5	0	5
**Unable**	1	0	1
Q04 Pain*			
**No pain or discomfort**	33	28	5
**Slight pain or discomfort**	17	7	10
**Moderate pain or discomfort**	11	0	11
**Severe pain or discomfort**	7	0	7
**Extreme pain or discomfort**	0	0	0
Q05 Anxiety / Depression*			
**Not worried, sad, or unhappy**	46	33	13
**Slightly worried, sad, or unhappy**	16	2	14
**Moderately worried, sad, or unhappy**	5	0	5
**Severely worried, sad, or unhappy**	1	0	1
**Extremely worried, sad, or unhappy**	0	0	0
EQ-VAS (0–100)**			
**median (IQR)**	81.5 (69.3–96.5)	96 (89–100)	70 (40–76)
EQ-5D Sum Score (5–25)**			
**median (IQR)**	6 (5–10)	5 (5–6)	10 (7–14)

*p-value < 0.001 by Fisher’s exact when responses were dichotomized into ‘no problems’ vs. ‘any problems’

**p-value < 0.001 by Mann-Whitney U test

cJADAS-71: clinical juvenile arthritis disease activity score with 71 joint count; EQ-5D-Y-5L: EuroQol five-dimensional ‘youth’ questionnaire with five levels; IQR: interquartile range

**Fig. 1:**
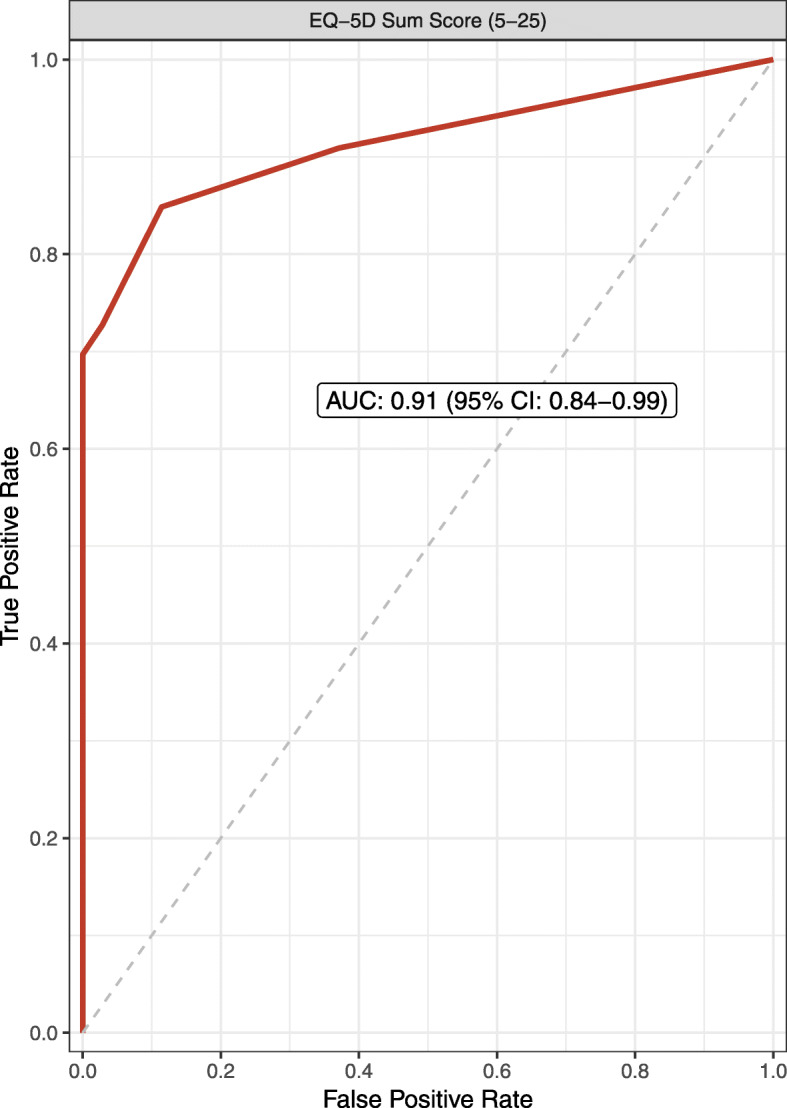
Discriminatory power of the EQ-5D-Y-5L sum score to identify moderate to high disease activity. AUC: area under the curve

ROC curves demonstrated the discriminatory power of the EQ-5D sum score (AUC 0.91, 95% CI 0.84–0.99) regarding identification of JIA patients with moderate to high disease activity (Fig. 1). ROC curves of individual EQ-5D-Y-5L domains and EQ-VAS are presented in the Supplementary Info (See Supplementary Figures S8A-F, Additional File 1)*.*

Optimal thresholds were identified using Youden’s Index. The EQ-5D sum score and EQ-VAS displayed comparable diagnostic accuracy (87%). Full diagnostic characteristics are presented in Table 3.

**Table 3:** Discriminatory power of EQ-5D-Y-5L thresholds to identify JIA patients with moderate to high disease activity

EQ-5D-Y-5L Thresholds	Accuracy (%)	95% CI	Sensitivity (%)	Specificity (%)	PPV (%)	NPV (%)
**EQ-5D Sum Score > 6**	87^*^	76–94	85	89	88	86
**EQ-VAS < 82**	87^*^	76–94	88	86	85	88
**Mobility > 1**	84^*^	75–93	70	97	96	77
**Self-Care > 1**	72^*^	60–88	42	100	100	65
**Daily Activities > 1**	79^*^	68–88	82	77	77	82
**Pain/Discomfort > 1**	82^*^	71–91	85	80	80	85
**Anxiety/Depression > 1**	78^*^	66–87	61	94	91	72

^*^P-value < 0.001; EQ-5D-Y-5L: EuroQol five-dimensional ‘youth’ questionnaire with five levels; EQ-5D sum score: unweighted sum of EQ-5D-Y-5L individual level scores (range 5–25); EQ-VAS: EuroQol visual analogue scale (range 0–100); NPV: negative predictive value; PPV: positive predictive value

## Discussion

The EQ-5D-Y-5L completed via the Reuma2Go application was able to identify JIA patients with moderate to high disease activity in need of possible treatment adjustments. The EQ-5D sum score could identify these patients with satisfactory accuracy, sensitivity and negative predictive value. Thus far, our data indicate that disease activity requiring treatment changes would not have been missed using the identified thresholds. Therefore, the Reuma2Go application could provide physicians with important information without the requirement of a clinical visit. This could be especially useful in regions or situations where regular out-patient clinic visits are not feasible.

Future research should elaborate on the safety of remote monitoring and optimizing visit frequency of JIA patients in remission on medication, as well as the cost-effectiveness of such interventions. Long-term usage of E-health applications and their effect on self-management, disease assessment and physician-patient interaction will be investigated. Also, addition of the identity of the respondent (parent or patient) may further improve the results. Recent studies on interactive technologies to promote disease self-management have demonstrated feasibility and initial results warrant future research of such interventions [24, 25].

Our results are subject to limitations connected to a population with overall low disease activity and a relatively small sample size. This forced us to dichotomize the answers into “no problems” and “any problems”, and prevented further sub-group analyses of patients that indicated any problems. Prolonged data collection is necessary to investigate the responsiveness and reliability of the EQ-5D-Y-5L. These results confirm the relationship of HRQoL with disease severity found in previous studies [11, 26, 27].

## Conclusions

In summary, initial results illustrate the value of self-assessment and E-health applications for remote monitoring of patients with JIA. Monitoring of PROs and HRQoL through smart devices could revolutionize information collection and contribute to a continuum of care for patients with JIA. Simultaneously, this provides physicians with important information to determine the frequency of clinical visits, assess therapeutic efficacy and guide treat-to-target strategies.

## Supplementary Information

**Additional file 1.** Information and screenshots on the EQ-5D-Y-5 L questionnaire as presented in the Reuma2go mobile application. ROC curves of individual EQ-5D-Y-5 L domains and EQ-VAS to identify JIA patients with moderate to high disease activity.

## Data Availability

The datasets used and/or analysed during the current study are available from the corresponding author on reasonable request.

## Abbreviations

AJC: Active Joint Count
AUC: Area Under the Curve
cJADAS-71: clinical Juvenile Arthritis Disease Activity Score with 71 joint count
EQ-5D-Y-5 L: EuroQol five-dimensional ‘Youth’ questionnaire with five levels
HRQoL: Health-related Quality of Life
JAMAR: Juvenile Arthritis Multidimensional Assessment Report
JIA: Juvenile Idiopathic Arthritis
NPV: Negative Predictive Value
PGA: Physician Global Assessment
PPV: Positive Predictive Value
PRO: Patient-reported Outcome
ROC curve: Receiver Operating Characteristic curve
VAS: Visual Analogue Scale
