# Supplementary material for: Monitoring patients with juvenile idiopathic arthritis using health-related quality of life
Source: Pediatr Rheumatol Online J. 2021 Mar 22;19:40. doi: 10.1186/s12969-021-00527-z (PMC7986307; doi:10.1186/s12969-021-00527-z)
Supplement: Supplementary file 1 — Additional file 1. Information and screenshots on the EQ-5D-Y-5 L questionnaire as presented in the Reuma2go mobile application. ROC curves of individual EQ-5D-Y-5 L domains and EQ-VAS to identify JIA patients with moderate to high disease activity. [file 12969_2021_527_MOESM1_ESM.pdf]

Additional File 1

Reuma2Go

'Reuma2Go' is a mobile E-health application for the clinical care of children and adolescents with juvenile idiopathic arthritis (JIA) developed in collaboration with patients, parents and caregivers.

The mobile application provides patients with a mobile interface for the self-assessment of joint pain, morning stiffness, usual activities and fatigue, and operates as a communication platform between patients and health care professionals.

In addition, the EQ-5D-Y-5L is included as measurement of health-related quality of life (see screenshots below). Data from the Reuma2Go application are collected in real-time and digitally monitored by our medical staff using a web-based portal.

Supplementary Figure S1.
EQ-5D-Y-5L Questionnaire as displayed in the Reuma2go application (Step 1 of 7).

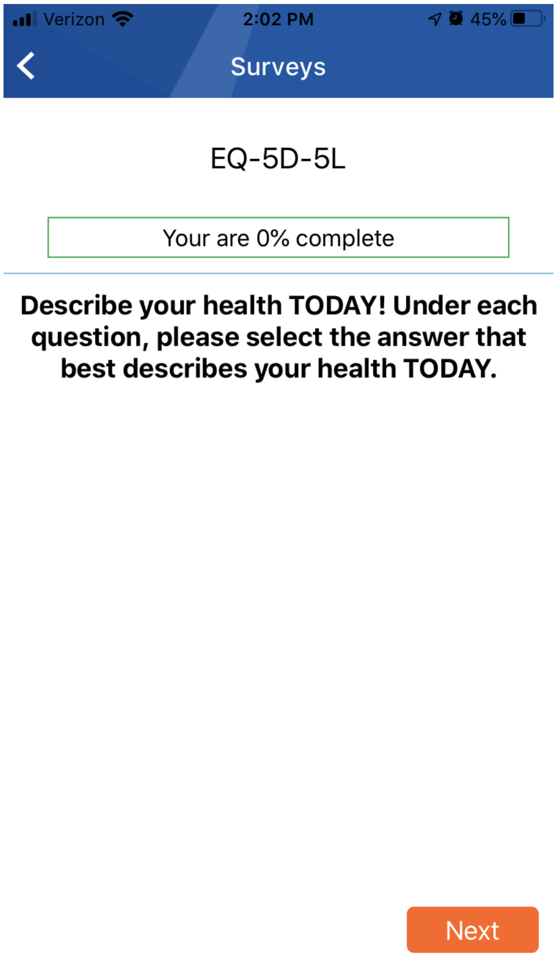

Supplementary Figure S2.
EQ-5D-Y-5L Questionnaire as displayed in the Reuma2go application (Step 2 of 7).

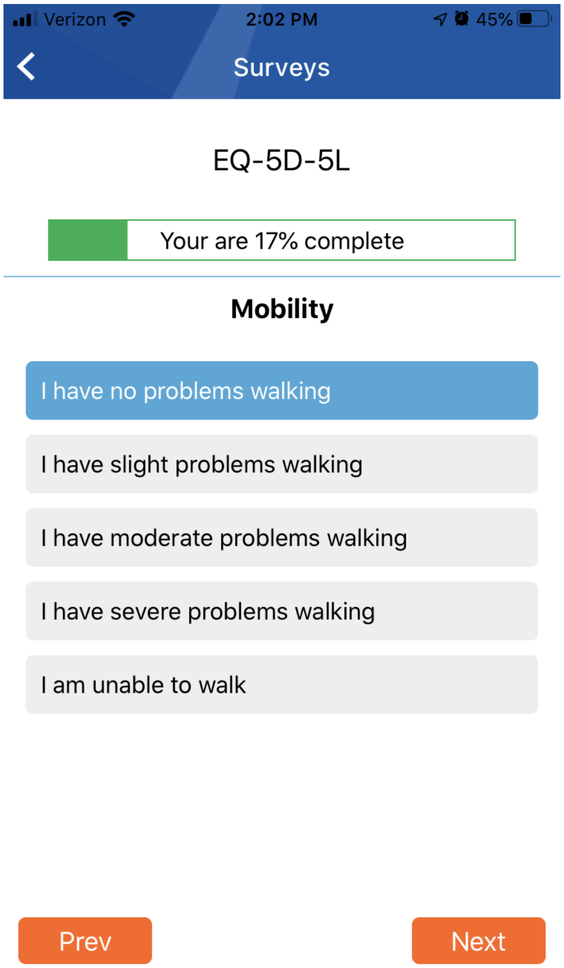

Supplementary Figure S3.
EQ-5D-Y-5L Questionnaire as displayed in the Reuma2go application (Step 3 of 7).

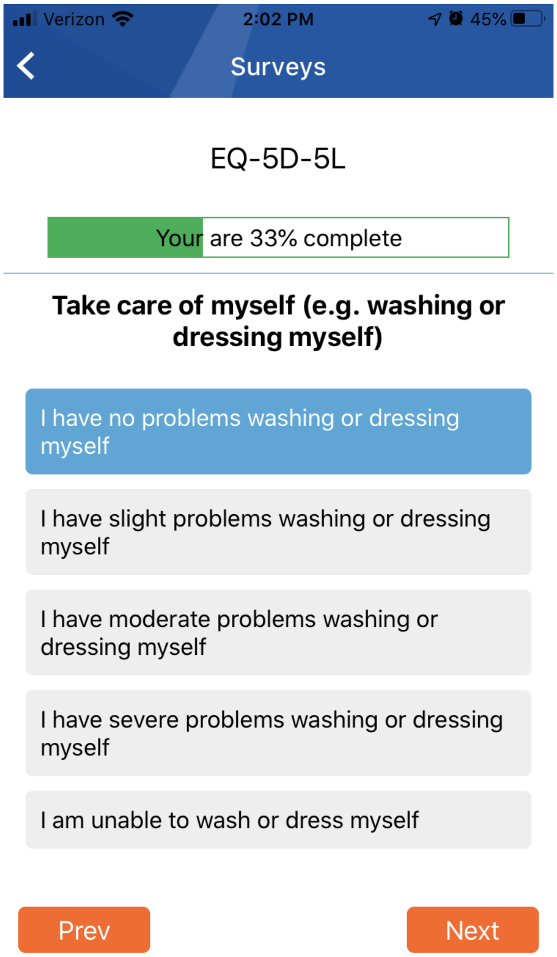

Supplementary Figure S4.
EQ-5D-Y-5L Questionnaire as displayed in the Reuma2go application (Step 4 of 7).

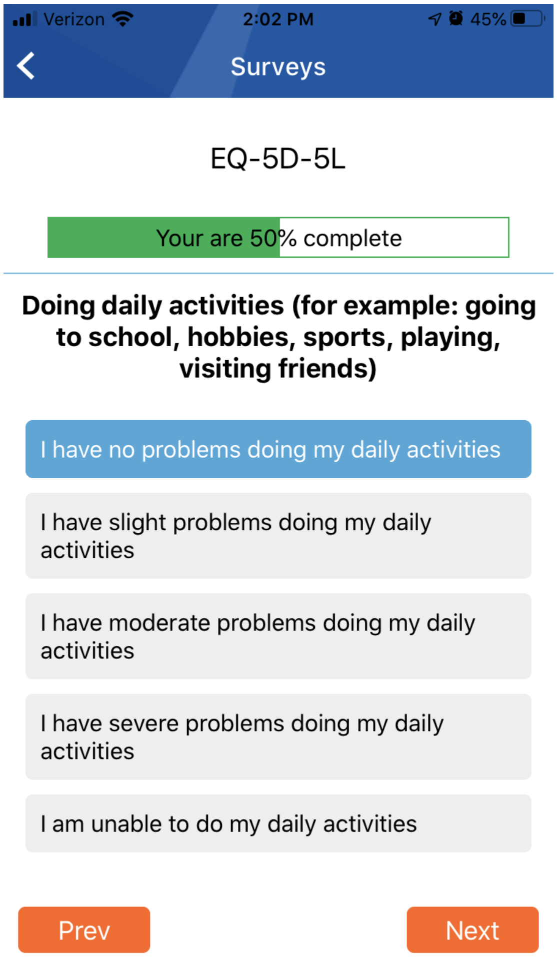

Supplementary Figure S5.
EQ-5D-Y-5L Questionnaire as displayed in the Reuma2go application (Step 5 of 7).

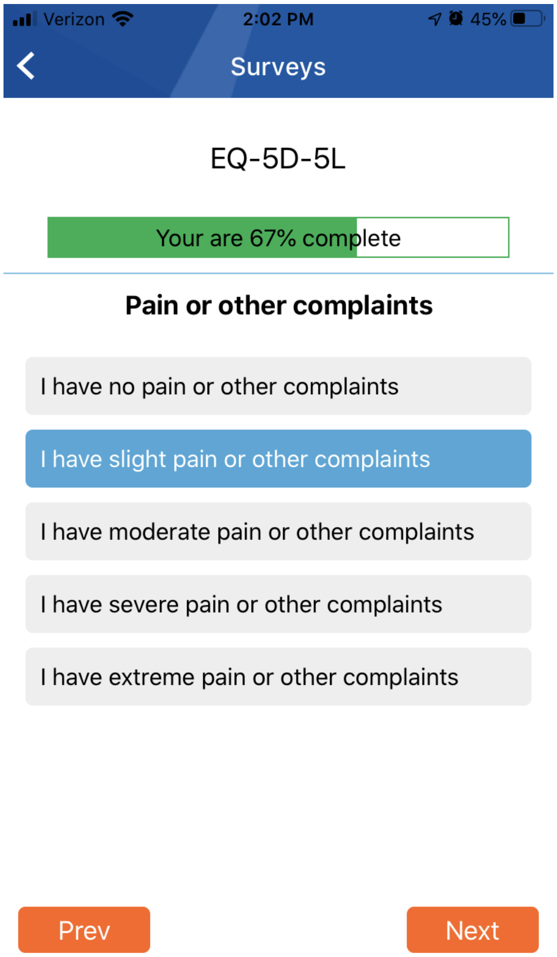

Supplementary Figure S6.
EQ-5D-Y-5L Questionnaire as displayed in the Reuma2go application (Step 6 of 7).

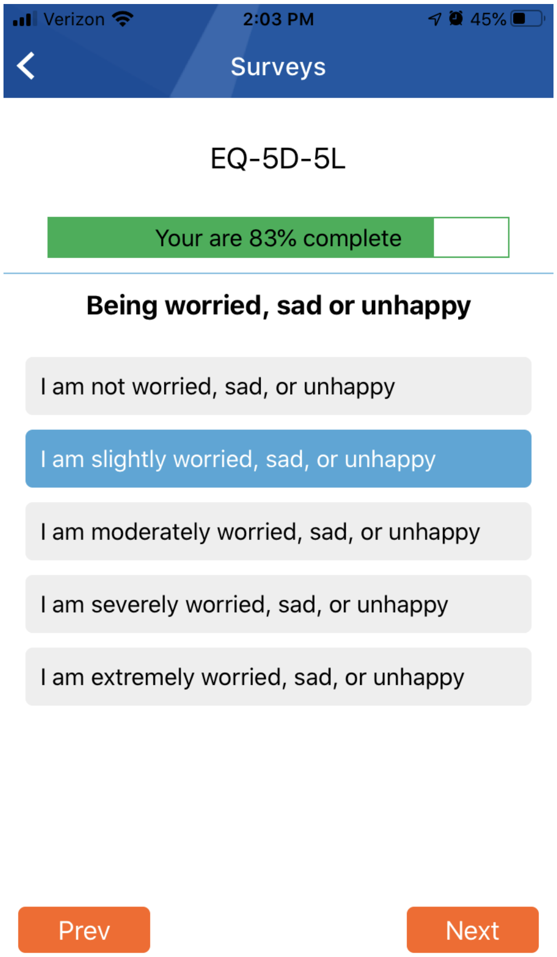

Supplementary Figure S7.
EQ-5D-Y-5L Questionnaire as displayed in the Reuma2go application (Step 7 of 7).

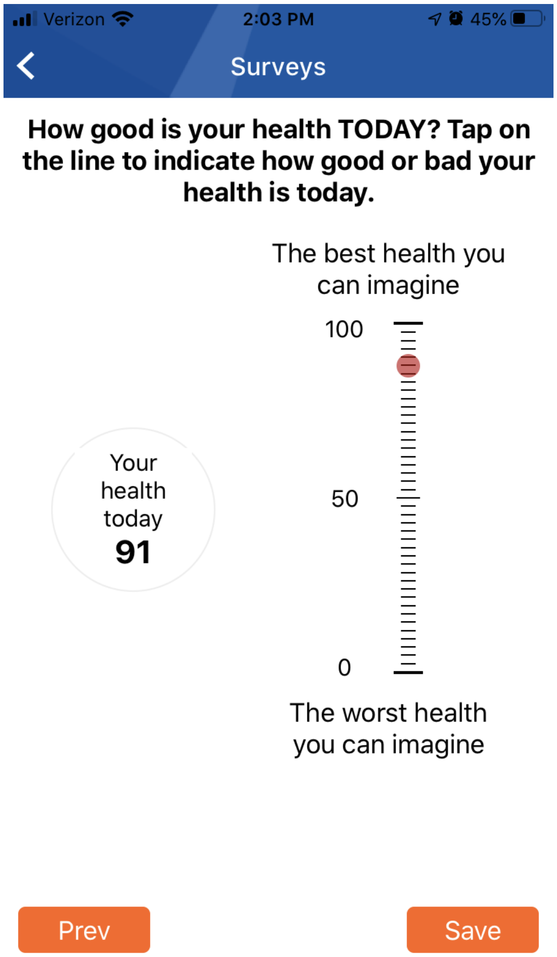

Supplementary Figure S8A-F. ROC curves of individual EQ-5D-Y-5L domains and EQ-VAS to identify JIA patients with moderate to high disease activity.

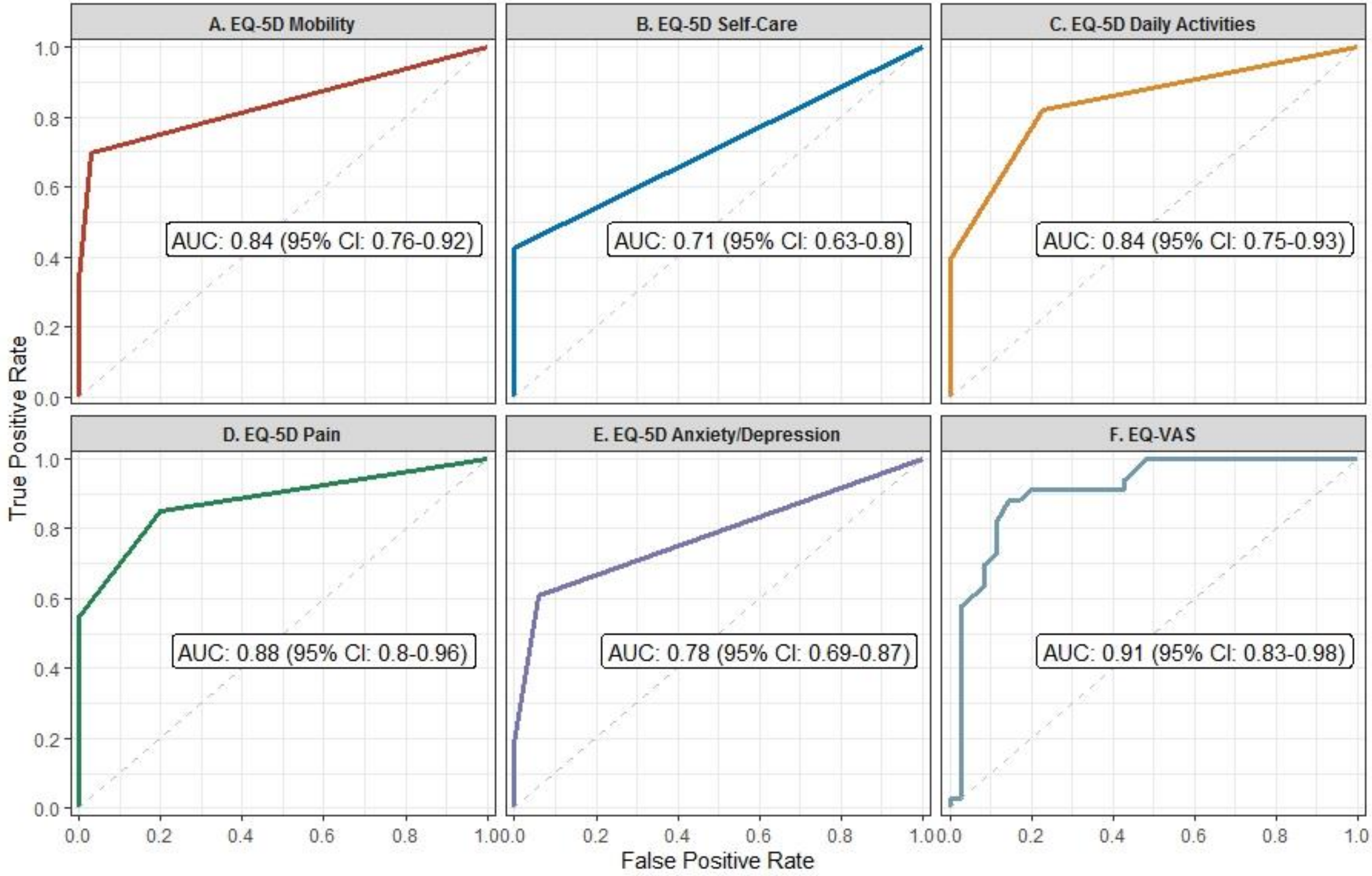

Acknowledgements: The EQ-5D-Y-5L is a copyrighted instrument, which can be obtained from the EuroQol Research Foundation by registering at their website www.euroqol.org. No license fee will be charged for non-commercial use.

Reuma2Go is a copyrighted mobile application and accompanying web-based portal was built on the MyOwnMed, Inc., platform (Bethesda, USA) MyOwnMed, Inc.
